# Ultrasound-guided infraclavicular axillary vein access for central venous catheterization: an evidence-informed narrative review

**DOI:** 10.3389/fmed.2026.1999229

**Published:** 2026-10-28

**Authors:** Dan Li, Xueyi Shang, Liyuan Ren, Yaqi Tian, Guoqing Zhang, Le Gao

**Affiliations:** 1 Senior Department of Oncology, Chinese People’s Liberation Army General Hospital, Beijing, China; 2 Department of Critical Care Medicine, The Fifth Medical Center of Chinese PLA General Hospital, Beijing, China

**Keywords:** axillary vein, catheter-related infection, central venous catheterization, clinical competence, infraclavicular access, thrombosis, ultrasonography, vascular access devices

## Abstract

This evidence-informed narrative review addresses a longstanding anatomical inconsistency between the axillary vein (AXV) and subclavian vein (SCV), whereby some studies labeled as ultrasound-guided ’subclavian' catheterization actually puncture the AXV lateral to the first rib. We integrate first-rib-based anatomy, preprocedural ultrasound assessment, puncture strategy, device selection, guidewire and catheter-tip management, comparative outcomes, complications, training, and implementation across the complete infraclavicular AXV access pathway. PubMed/MEDLINE, Embase, and Web of Science Core Collection were searched from inception through August 6, 2026, supplemented by guideline searches and citation tracking. Direct randomized evidence and a recent meta-analysis suggest that, in selected patients and trained operators, short-term overall catheterization success and early mechanical outcomes are broadly similar to internal jugular access, although the available evidence does not independently establish formal equivalence. Proximal targeting and arm-position optimization may improve procedural efficiency in selected settings, but effects vary with respiratory state, anatomical definition, side, and operator technique. Current evidence does not establish a long-term infection or thrombosis advantage or a universally superior ultrasound imaging plane. Guidewire and catheter malposition after successful venipuncture further show that puncture success alone is an incomplete safety endpoint. Infraclavicular AXV access should therefore be considered one of several potential first-line options in appropriately selected patients and trained teams, not a universal replacement for other central venous routes. We propose an externally unvalidated implementation framework linking nomenclature, dynamic target selection, needle-tip control, guidewire navigation, tip confirmation, immediate complication exclusion, securement, and maintenance.

## 1 Introduction

Central venous access is fundamental to critical care, perioperative medicine, oncology, and long-term intravenous therapy. The internal jugular vein (IJV) has remained a dominant site because it is readily visualized with ultrasound, compressible, and supported by a mature training pathway. Traditional subclavian vein (SCV) access has been valued for lower catheter-related infection rates and, in some settings, lower thrombosis risk, but landmark-guided puncture carries concerns about pneumothorax, hemothorax, and non-compressible arterial injury (1, 2). As real-time ultrasound has moved from an adjunct to a core safety tool for central venous catheterization, the identity of the vein actually visualized and punctured in the infraclavicular window requires more precise definition.

From a strict anatomical perspective, the AXV becomes the SCV at the lateral border of the first rib. A substantial portion of the true SCV lies behind the acoustic shadow of the clavicle, whereas the vein that is clearly visualized and punctured in real time through many infraclavicular ultrasound windows lies lateral to the first rib and is therefore the AXV (3–7). LeDonne, Biasucci and colleagues, and more recently Annetta and colleagues have emphasized that indiscriminate use of the term 'subclavian vein' for this target can cause anatomical misclassification and distort evidence synthesis (4, 5, 7). Accordingly, this review uses ‘infraclavicular axillary vein’ as the primary English term.

The evidence base has expanded from feasibility series to randomized comparisons of proximal and distal AXV targets, direct AXV-versus-IJV trials, studies of ultrasound planes and arm position, chest-wall totally implantable venous access devices (TIVADs), learning-curve studies, and a 2026 systematic review with trial sequential analysis (8–14). Important gaps remain. Short-term technical outcomes predominate, while long-term infection, thrombosis, device survival, and patient-centered outcomes are less well characterized. Definitions of ‘proximal AXV,’ ‘distal AXV,’ and ‘infraclavicular SCV’ vary, and several comparisons simultaneously change both anatomical target and imaging strategy, making it difficult to isolate the effect of access site itself.

The 2026 meta-analysis primarily addressed overall catheterization success and early mechanical safety for AXV versus IJV in critically ill patients (12), whereas recent terminology work focused on AXV/SCV confusion (7). The added value of the present review is to reclassify evidence according to the first-rib boundary, distinguish anatomical directness from study design and potential bias, and integrate puncture, guidewire navigation, catheter advancement, tip location, and maintenance as one complete access pathway. The central question is therefore not simply whether infraclavicular AXV access is feasible, but how it should be positioned within modern ultrasound-guided central venous access and which conclusions are supported by direct versus extrapolated evidence.

## 2 Review methods

### 2.1 Review design and scope

This was a targeted, reproducible, evidence-informed narrative review rather than a systematic review or meta-analysis. A narrative approach was selected because the relevant literature spans heterogeneous anatomical studies, technical reports, randomized trials, observational device cohorts, guidelines, and adjacent central-venous-access evidence, for which a single quantitative synthesis would not address the full clinical pathway. Review planning and reporting were informed by the Scale for the Assessment of Narrative Review Articles (SANRA) (15). Prespecified questions addressed anatomical definition, preprocedural target selection and positioning, puncture technique, device pathways, guidewire and catheter-tip management, comparative outcomes, complications, training, and clinical implementation.

### 2.2 Information sources and search strategy

PubMed/MEDLINE, Embase, and Web of Science Core Collection were searched from database inception through August 6, 2026, without language restriction. The search combined three concept blocks: AXV and infraclavicular/proximal/distal terminology; central venous catheterization and relevant device pathways; and ultrasound or real-time imaging. PubMed yielded 162 records, Embase yielded 302 platform hits (273 article records and 29 ClinicalTrials.gov registry records), and Web of Science Core Collection yielded 163 records. The 29 registry records were separated before bibliographic deduplication, leaving 598 bibliographic records. Vascular-access guidelines and consensus documents were also sought, and backward and forward citation tracking was performed for key reviews and primary studies. No separate systematic search of preprint servers or grey-literature repositories was undertaken; database-indexed conference records were screened under the same eligibility rules. Language was not used as an exclusion criterion. Exact search strings, search limits, dates, and database record counts are provided in Supplementary Table S1.

### 2.3 Deduplication, screening, and evidence selection

Duplicate detection used DOI, PMID, and normalized title plus first-author fields; conference supplements in which distinct abstracts shared a DOI were manually checked to avoid erroneous merging. Deduplication removed 267 records, leaving 331 unique records. Two reviewers independently performed structured title/abstract screening using prespecified rules; rule-based scoring served only as a triage aid and did not automatically exclude records. Disagreements during title/abstract screening were adjudicated by a third reviewer. Title/abstract screening excluded 148 records, leaving 183 records for secondary relevance and evidence-role assessment. The same two reviewers independently assessed these retained records using four structured domains: clinical relevance, anatomical directness, technical transferability, and redundancy. Clinical relevance reflected the extent to which a report informed infraclavicular AXV central venous access, relevant device pathways, clinically important outcomes, or implementation. Anatomical directness reflected whether the venous target could be localized using the lateral border of the first rib, an explicit infraclavicular location, ultrasound images, or another reproducible anatomical landmark. Technical transferability referred to whether findings from an adjacent vascular-access, device, or procedural setting could reasonably inform one or more components of the infraclavicular AXV access pathway. Redundancy was considered when the same clinical or technical point was already represented by a more direct, complete, or methodologically informative source. No single domain alone automatically determined retention. Records were retained in the core evidence matrix when they made a substantive, non-redundant contribution to the review and provided sufficient source detail to support the intended use of the evidence. Disagreements between the two reviewers at this secondary assessment stage were adjudicated by a third reviewer. Of the 183 records, 52 were retained as core database-derived evidence documents and 131 were not selected for the core evidence matrix; one primary disposition category was assigned to each of these 131 records (Supplementary Table S3). This secondary assessment was designed for evidence selection within a narrative review and was not equivalent to a systematic-review full-text eligibility stage. A separate supplementary/contextual pathway identified 21 documents through citation tracking, guideline searches, or broader central-venous-access context. During revision, three additional contextual documents were added to address reviewer-identified methodological and clinical issues. The final evidence portfolio therefore comprised 76 documents: 52 core-search and 24 supplementary/contextual documents. The evidence identification and selection process is summarized in Figure 1. Verification level was recorded source by source as full article, formal abstract, or metadata. Full articles were required when procedural details, numerical outcome data, or anatomical classification depended on information beyond the bibliographic record. Formal abstracts were used only for information explicitly reported in the abstract. Metadata-only records were not used to support procedural details, numerical outcome estimates, or anatomical reclassification. Verification status and claim boundaries for retained evidence sources are reported in Supplementary Table S2.

**Figure 1:**
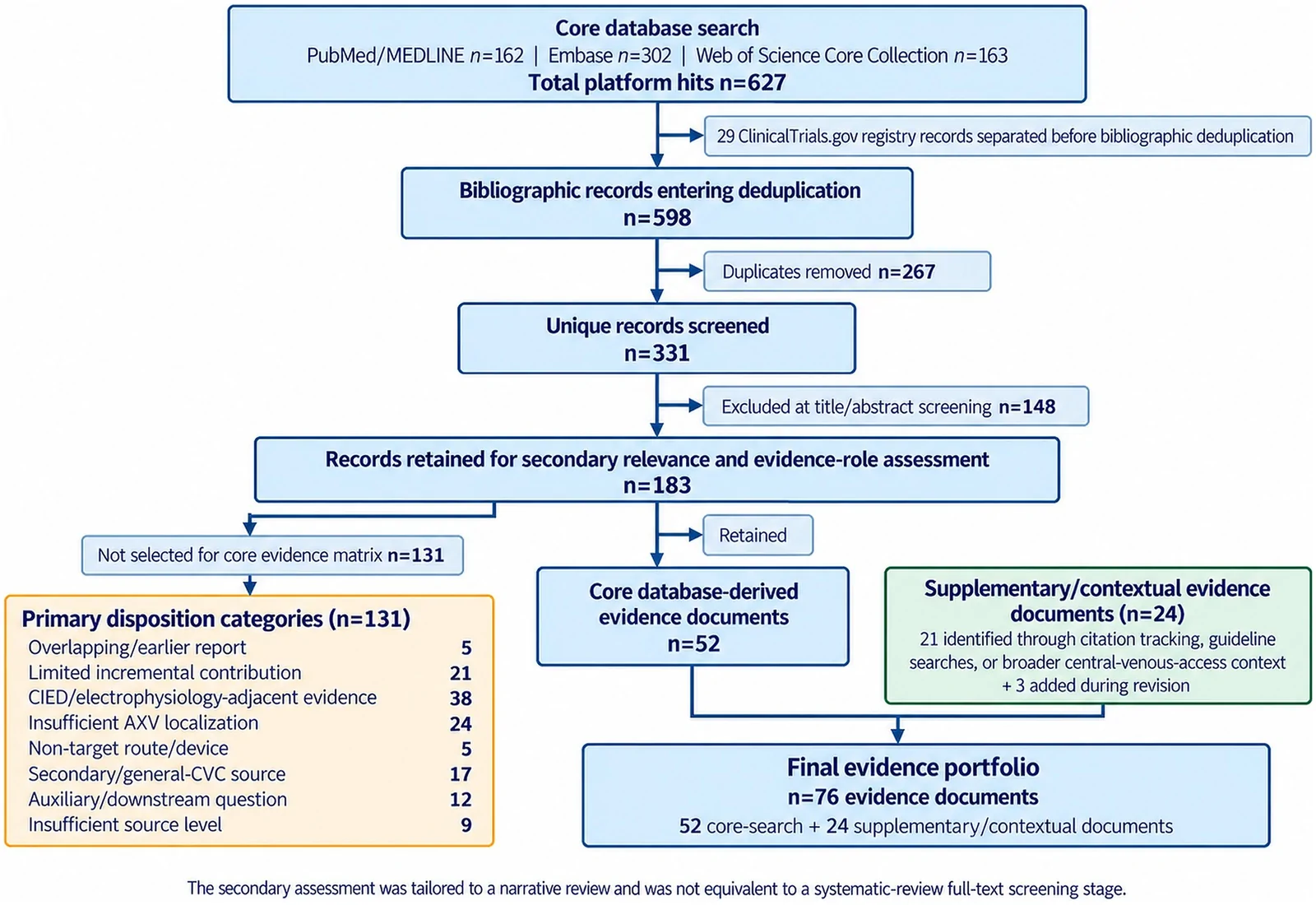
Evidence identification and selection flow for the evidence-informed narrative review. Three database platforms yielded 627 hits. Twenty-nine ClinicalTrials.gov registry records returned through Embase were separated before bibliographic deduplication, leaving 598 bibliographic records. After removal of 267 duplicates, 331 unique records underwent independent title/abstract screening by two reviewers; 148 were excluded and 183 proceeded to secondary relevance and evidence-role assessment by the same two reviewers, with disagreements at either stage adjudicated by a third reviewer. Fifty-two database-derived documents were retained in the core evidence matrix, while 131 were not selected for the core matrix after assessment of relevance, anatomical directness, technical transferability, and redundancy. Twenty-one supplementary/contextual documents were identified through citation tracking, guideline searches, or broader central-venous-access context, and three additional contextual documents were added during revision, yielding 76 final evidence documents. This process was tailored to an evidence-informed narrative review and was not equivalent to a systematic-review full-text eligibility stage.

Each retained evidence document was coded on two independent axes. The evidence-directness axis classified sources as D (direct AXV clinical evidence), T (anatomical/technical rationale), E (extrapolated evidence from general central venous access, mixed AXV/SCV populations, or adjacent techniques), or C (consensus/guideline context). The evidence-function axis recorded the source's contribution to anatomy/terminology, preprocedural assessment, puncture technique, device pathway, guidewire/tip management, comparative outcomes, complications, training, or implementation. Study-level coding, available Screen_ID cross-mapping, verification status, and claim boundaries are reported in Supplementary Table S2.

### 2.4 Eligibility boundaries, evidence classification, and synthesis

Eligible evidence included human clinical studies, healthy-volunteer or anatomical imaging studies, AXV-specific simulation or training research, systematic reviews, guidelines, and consensus documents. The target vessel had to be an ultrasound-guided infraclavicular AXV, proximal or distal AXV, or an ‘infraclavicular SCV’ that could be reasonably reinterpreted as AXV from reproducible anatomical information. Devices included CICCs, tunneled catheters, TIVADs, dialysis catheters, and selected technically transferable cardiac-device access studies. Exclusions included axillary-artery studies, studies confined to thrombosis diagnosis or arteriovenous-fistula surveillance, peripheral/midline/PICC studies without direct AXV evidence, landmark- or venography-only studies without a relevant ultrasound comparator, and reports without extractable evidence. Studies labeled only as 'subclavian,' ‘AxV/SCV,’ or ‘axillary’ without the first rib, infraclavicular location, or another reproducible landmark were not automatically treated as direct AXV evidence. For source-level anatomical classification, we prioritized the reported relationship between the puncture site and the lateral border of the first rib and additionally considered explicit infraclavicular localization, ultrasound images, and reproducible procedural descriptions. When these features were insufficient to establish the venous segment with confidence, the source was classified as indirect or adjacent evidence rather than being reclassified as direct AXV evidence. The source-specific basis for anatomical classification or reclassification is reported in Supplementary Table S2.

Evidence directness was not treated as an evidence-quality grade. Study design, sample size, outcome definitions, follow-up, major limitations, and the claims each source could support were recorded separately. Evidence was synthesized qualitatively; no new meta-analysis, formal risk-of-bias tool, GRADE assessment, or PROSPERO registration was undertaken.

## 3 Anatomical boundaries and nomenclature

### 3.1 The first-rib boundary between the AXV and SCV

By conventional anatomy, the AXV becomes the SCV at the lateral border of the first rib. The lateral border of the first rib, rather than the clavicle itself, is therefore the most reproducible anatomical landmark separating the two venous segments (4–7, 16–18). With real-time ultrasound, the vein can be followed from lateral to medial as the AXV approaches the first rib and then enters the region of clavicular acoustic shadow. If the puncture site is lateral to the first rib, the target is anatomically more consistent with the AXV even when a study describes 'subclavian access’ or ‘infraclavicular SCV.’

Terminology is not merely semantic. If AXV puncture is classified as SCV access, its success and mechanical complication rates may be incorrectly assigned to the SCV literature. Conversely, extrapolating infection or thrombosis advantages from traditional landmark-guided SCV studies directly to the AXV produces the opposite form of misclassification (5, 7, 12). We therefore prioritized the relationship between the puncture site and first rib, the ultrasound images, and the procedural description when judging evidence directness.

### 3.2 Relationship among infraclavicular, proximal, and distal AXV terminology

‘Infraclavicular AXV’ denotes the AXV lateral to the first rib that is accessible through an infraclavicular ultrasound window. ‘Proximal AXV’ generally refers to a target closer to the AXV-SCV transition, but study-specific definitions differ; it should not be treated as automatically synonymous with the entire infraclavicular AXV (8, 9, 19). Galloway and Bodenham demonstrated that the AXV changes continuously along the infraclavicular course rather than at a natural proximal/distal dichotomy: medially the vein tends to be larger and more superficial but closer to the artery and thoracic cage, whereas laterally it becomes smaller and deeper, with less arterial overlap and greater distance from the thorax (3). This review therefore retains each study's operational proximal/distal definition rather than imposing a new universal cutoff.

The terminology and device-classification framework used throughout this review is summarized in Table 1. Figure 2 illustrates the first-rib boundary and candidate infraclavicular AXV targets.

**Table 1:** Terminology and device-classification framework.

Term	Working definition used in this review	Recommended English expression	Misuse to avoid
Axillary vein (AXV)	Axillary vein lateral to the lateral border of the first rib	Axillary vein	Using the clavicle itself as the AXV/SCV boundary
Subclavian vein (SCV)	Central continuation of the AXV after it crosses the lateral border of the first rib	Subclavian vein	Calling every vein visible in the infraclavicular ultrasound window the SCV
Infraclavicular AXV	AXV lateral to the first rib that can be visualized and punctured in real time through an infraclavicular ultrasound window	Infraclavicular axillary vein	Using ‘thoracic axillary vein’ as the primary English term
Proximal AXV	Study-defined AXV target closer to the first rib/central direction	Proximal axillary vein	Assuming all studies use the same proximal boundary
CICC	Central catheter inserted through a deep central vein such as the IJV, AXV, or SCV	Centrally inserted central catheter	Calling a catheter inserted directly through the AXV a PICC
Tc-/Tnc-CICC	Tunneled cuffed/non-cuffed centrally inserted central catheter	Tunneled cuffed/non-cuffed CICC	Classifying the device according to skin-exit position rather than venous entry and device structure
Chest TIVAD	Device inserted through a deep central vein and connected to a subcutaneous chest reservoir	Chest-TIVAD/chest port	Using ‘Port-a-cath’ as the universal scientific device category

Device nomenclature should be determined primarily by venous entry site and device structure rather than by the skin exit site. The framework is aligned with the NAVIGATE nomenclature (34).

**Figure 2:**
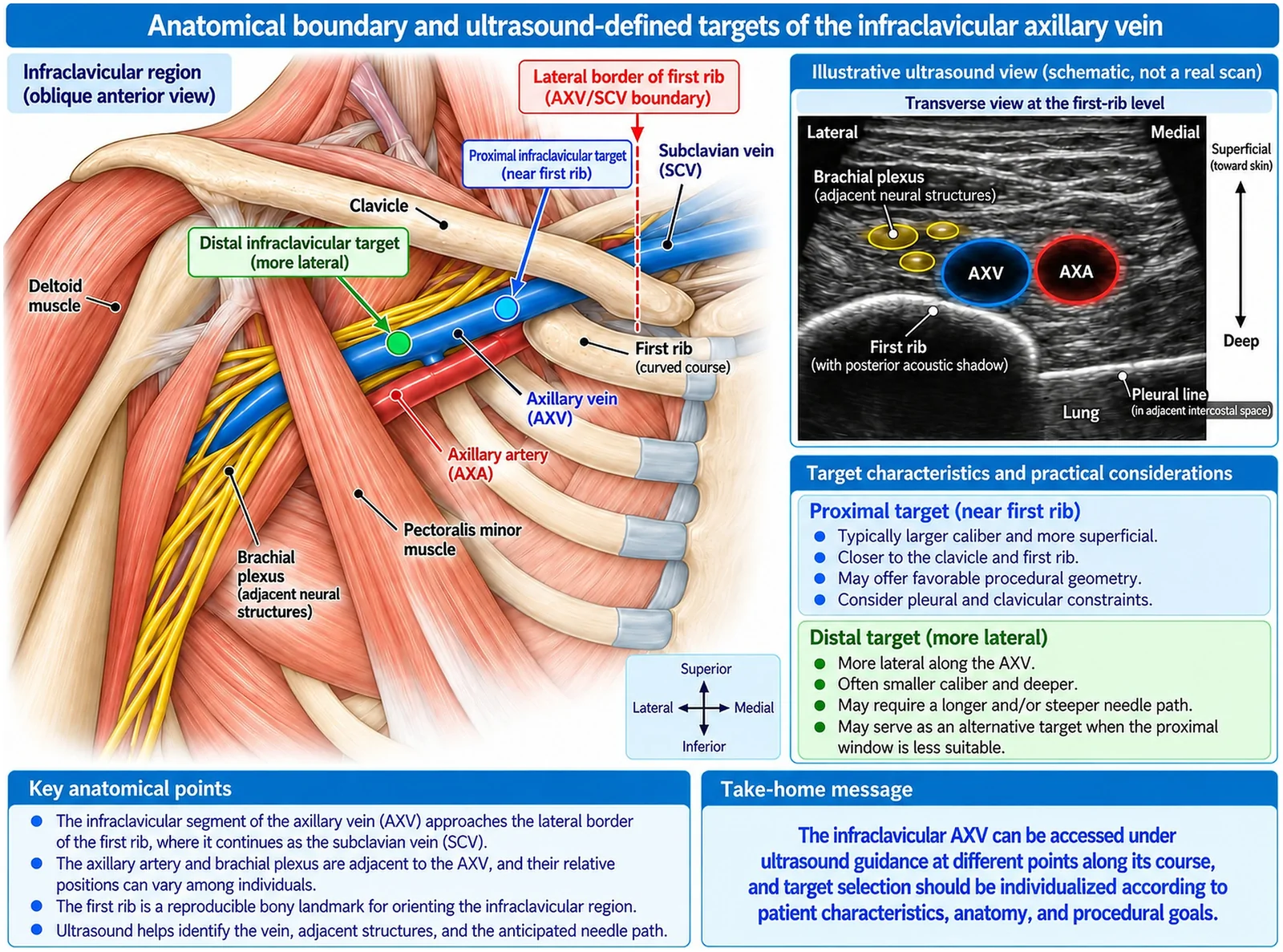
Anatomical boundary and ultrasound-defined targets of the infraclavicular axillary vein. The lateral border of the first rib marks the anatomical transition from the axillary vein (AXV) to the subclavian vein (SCV). The illustration shows candidate proximal and distal infraclavicular targets, the axillary artery, first rib, clavicle, brachial plexus, and pleural line. The ultrasound panel is schematic rather than patient-derived. Proximal and distal targets are alternative procedural windows rather than universally superior options; selection should be individualized according to vessel size, depth, compressibility, adjacent structures, and the anticipated needle path. AXA, axillary artery; AXV, axillary vein; SCV, subclavian vein.

## 4 Preprocedural ultrasound assessment and target selection

### 4.1 From finding a vein to evaluating an access window

Preprocedural ultrasound should do more than establish that a vein is present; it should determine whether the target forms a usable and safe access window. Frameworks such as Rapid Central Vein Assessment (RaCeVA) emphasize systematic scanning, venous patency, adjacent arteries and pleura, thrombosis, and pathway feasibility (20). For infraclavicular AXV access, assessment should include vessel diameter and depth, respiratory collapsibility, artery-vein overlap, distance to the pleura/chest wall, anticipated needle length and angle, and ipsilateral history of central lines, thoracic surgery, radiotherapy, thrombosis, or local infection.

These parameters trade off against one another. The largest vessel is not necessarily the optimal target, and the site most distant from the pleura may not yield the best catheterization efficiency. A practical target should permit stable vessel identification, sustained needle-tip control, an adequate safety margin from adjacent structures, and a reasonable route for guidewire and catheter passage into the central venous system.

### 4.2 Arm position and respiratory state

Arm abduction can alter the relationship between the clavicle and AXV and increase sonographic exposure of the proximal vein. Pittiruti and colleagues found that 90-degree arm abduction with shoulder protraction increased AXV diameter in most participants (21), and Ravindran and colleagues observed reduced respiratory collapsibility in spontaneously breathing patients (22). In a randomized trial of spontaneously breathing patients, Nagalingam and colleagues reported that 90-degree abduction increased first-pass guidewire success from 51.9% to 78.6%, reduced failure to achieve cannulation within the prespecified attempts in the assigned position from 26.9% to 0%, and reduced catheter-tip malposition from 15.8% to 1.5% (23).

However, the effect is context dependent. In a randomized trial of 60 mechanically ventilated patients, arm abduction moved the clavicle cephalad by 2.2 ± 0.6 cm, exposed an additional 2.2 ± 0.5 cm of proximal AXV, and increased the vein-to-pleura distance from approximately 6 to 9 mm, yet first-pass success was 24/30 (80.0%) versus 21/30 (70.0%; *p* = 0.371) and overall success was 28/30 (93.3%) versus 29/30 (96.7%; *p* = 0.554), with no arterial puncture or pneumothorax in either group (24). In Ahn and colleagues' 481-patient randomized trial of right proximal AXV catheterization, 90-degree abduction did not improve completion of catheterization (238/241 vs. 233/240; *p* = 0.199) but reduced catheter malposition (1/238 vs. 9/233; *p* = 0.010) (25). Thus, the most consistent benefit of abduction is improved anatomical exposure and, in some settings, better guidewire/catheter pathway outcomes rather than a universal increase in puncture or overall success. Positioning should therefore be treated as a dynamic optimization variable influenced by respiratory state, side, shoulder mobility, and operator factors.

### 4.3 Proximal versus distal targets

Randomized comparisons suggest that proximal targeting may improve procedural efficiency, although findings are not uniform across studies. Wang and colleagues reported in 99 older patients that the proximal AXV had a diameter of 0.80 ± 0.33 cm versus 0.61 ± 0.33 cm distally and a collapsibility index of 20% ± 27% versus 56% ± 34%; first-pass success was 71.4% versus 42.0% and overall success was 79.6% versus 54.0% (8). In the PANDA trial, first-pass success was 75.8% versus 51.5% and site success was 93.9% versus 83.8% for proximal versus distal access; overall success was 99% in both groups, but proximal access required fewer attempts and shorter access and cannulation times (9).

Buzançais and colleagues compared distal with proximal AXV access in 119 analyzable patients. Initial allocated-approach success was 85.5% versus 87.7% (distal minus proximal risk difference −2.2 percentage points; 90% CI −12.5 to 8.1), and the prespecified −8 percentage-point non-inferiority criterion was not met. Overall success after crossover was 98.4% versus 96.5%; at the overall strategy level, the distal-start strategy met the study's prespecified non-inferiority criterion, which should not be conflated with non-inferiority of the initially allocated distal approach. Catheter-tip positions considered to confer greater thrombotic risk were more frequent after distal access (31.7% vs. 12.3%), but this was a surrogate tip-position endpoint rather than observed clinical catheter-related thrombosis (19). Current evidence therefore supports the narrower conclusion that proximal or mid-infraclavicular targets may improve first-pass success and procedural efficiency in selected patients; it does not establish a long-term infection, thrombosis, or device-survival advantage.

### 4.4 Catheter-to-vein ratio: no validated AXV-specific threshold

Catheter-to-vein ratio is used in PICC-related thrombosis prevention and is incorporated into contemporary vascular access guidance (26). However, this evidence audit identified no validated catheter-to-vein ratio threshold specific to infraclavicular AXV CICC placement. PICCs and AXV CICCs differ in vessel flow, vessel size, catheter type, entry angle, and patient population. Until AXV-specific prospective dose-response data are available, fixed thresholds derived from PICC practice should not be presented as evidence-based exclusion criteria for infraclavicular AXV access.

Practical preprocedural ultrasound assessment domains and their interpretive boundaries are summarized in Table 2.

**Table 2:** Preprocedural ultrasound assessment and target-selection factors for infraclavicular AXV access.

Assessment domain	Potentially favorable feature	Potential risk signal	Evidence and interpretation
Vessel size/depth	Adequate caliber and moderate depth	Very small or deep vessel; marked collapse with low volume status	Proximal targets are often larger and less collapsible; lateral targets may provide a greater safety margin in some patients (3, 8, 9)
Respiratory collapsibility	Stable lumen after positioning optimization in spontaneously breathing patients	Near-complete inspiratory collapse	Benefits of arm abduction appear more consistent in spontaneously breathing than mechanically ventilated patients (21–24)
Artery-vein relationship	Limited overlap and a needle path that can be continuously visualized	Marked overlap or anticipated needle path crossing an artery	Artery-vein overlap generally decreases from medial to lateral (3)
Pleura/chest-wall distance	Clearly identifiable separation between the projected needle path and pleural line	Pleura immediately adjacent to the target or poor visualization	Pleural identification should be incorporated into target selection and needle advancement (46, 72)
Guidewire pathway	Expected central course with ultrasound confirmation when feasible	Resistance, aberrant course, or entry into ipsilateral IJV	Guidewire course is a separate safety endpoint from venipuncture (9, 23, 27, 45)
Patient/device context	Target compatible with indication, device, dwell time, and maintenance plan	Ipsilateral thrombosis/obstruction, local infection, device-route mismatch	Selection should integrate anatomy, device category, and the complete access pathway (26, 34, 47)

AXV, axillary vein; IJV, internal jugular vein.

## 5 Ultrasound-guided puncture technique

### 5.1 Long-axis/in-plane: conceptual benefit of continuous needle-tip visualization

The long-axis/in-plane (LA/IP) approach displays a longer venous segment and the needle shaft and can facilitate continuous needle-tip tracking when alignment is maintained. In 202 mechanically ventilated ICU procedures using LA/IP, Czarnik and colleagues reported 98.5% venipuncture success, 84.1% first-pass success, and 95.1% complete catheterization success (95% CI 91.1-97.6), yet there were six guidewire failures, 27 catheter-tip malpositions, and one pneumothorax (27). High venipuncture success therefore does not imply an event-free complete pathway. LA/IP still requires the probe, vessel, and needle to remain co-planar; with deep targets or steep angles the shaft may be visible while the true tip is lost.

### 5.2 Short-axis/out-of-plane: easy anatomical orientation, but needle-tip confirmation is critical

The short-axis/out-of-plane (SA/OOP) approach can display the vein, artery, and adjacent structures in cross-section and is useful for rapid anatomical orientation. The needle, however, appears as a point-like echo. Dynamic advancement, tilting, or other tip-confirmation maneuvers are therefore required to distinguish the true tip from a cross-section of the shaft. Glen and colleagues nevertheless reported a 94% success rate with real-time OOP infraclavicular AXV cannulation in a complex mechanically ventilated ICU population (28).

### 5.3 Patient-level comparisons show that technique effects are context dependent

Patient-level comparisons have not established a universally superior ultrasound plane. In a retrospective study by experienced operators, He and colleagues found higher one-attempt success with LA/IP than SA/OOP [110/120 [91.7%] vs. 96/116 [82.8%], *p* = 0.040], but total procedure time was longer (287.5 ± 19.6 vs. 184.7 ± 8.1 s, *p* < 0.001) (29). Conversely, in Maddali and colleagues' randomized trial of 86 adult cardiac surgical patients undergoing left AXV cannulation, OOP achieved higher first-pass success than IP [34/43 [79.1%] vs. 20/43 [46.5%], *p* < 0.01], required fewer total cannulation attempts (1.4 ± 0.7 vs. 1.8 ± 0.9, *p* < 0.05), and had a shorter total procedure time (6.9 ± 3.8 vs. 10.3 ± 5.2 min, *p* < 0.01). Needle redirections did not differ significantly (1.3 ± 0.9 vs. 1.0 ± 1.15, *p* = 0.26), and differences in arterial puncture, hematoma, pneumothorax, and abandonment were not significant (10). Imaging-plane selection should therefore reflect anatomy, side, respiratory state, and the operator's ability to maintain needle-tip control rather than assuming that LA/IP or OOP is intrinsically best.

### 5.4 Oblique-axis/in-plane and short-axis/in-plane: alternative approaches with limited comparative evidence

The oblique-axis/in-plane (OA/IP) approach seeks to combine anatomical orientation with in-plane needle visualization. Brescia and colleagues reported 96% first-pass success in an 80-patient oncology chest-port series, but there was no concurrent comparator (30). Tang and colleagues described a short-axis/in-plane (SA/IP) technique in a two-page technical communication and reported that 33/40 initial cases (82.5%) achieved first-pass access without posterior-wall puncture, with no reported failures or complications (31). Because the report lacked complete eligibility criteria, operator/device detail, outcome definitions, follow-up, and statistical methods, it was treated as T-class technical rationale rather than formal clinical effect evidence. OA/IP and SA/IP are plausible options, but both need standardized head-to-head evaluation.

### 5.5 Echogenic needles and navigation aids do not obviate the need for needle-tip control

In a 100-patient randomized trial, Kåsine and colleagues found that an echogenic needle did not shorten venous access time or improve first-pass success or safety (32). In 2026, Boulet and colleagues compared magnetic needle tracking with standard ultrasound in a single-center randomized trial: uncomplicated first-puncture success was 47/57 (82.5%) versus 44/63 (69.8%), an absolute difference of +12.6 percentage points (*p* = 0.053), while the composite of failure or immediate complication occurred in 5/57 (8.8%) versus 14/63 (22.2%), an absolute difference of −13.5 percentage points (*p* = 0.044) (33). Because the first-rib boundary was not reported, this trial was interpreted as adjacent technology evidence; additionally, the primary endpoint was not statistically significant and the composite endpoint was heterogeneous (33). Needle-guidance devices may be promising, but they do not substitute for continuous confirmation of the needle tip during the critical phases of advancement.

Figure 3 summarizes the four principal ultrasound view/needle-trajectory combinations and emphasizes that complications related to poor needle-tip control are not unique to a single plane.

**Figure 3:**
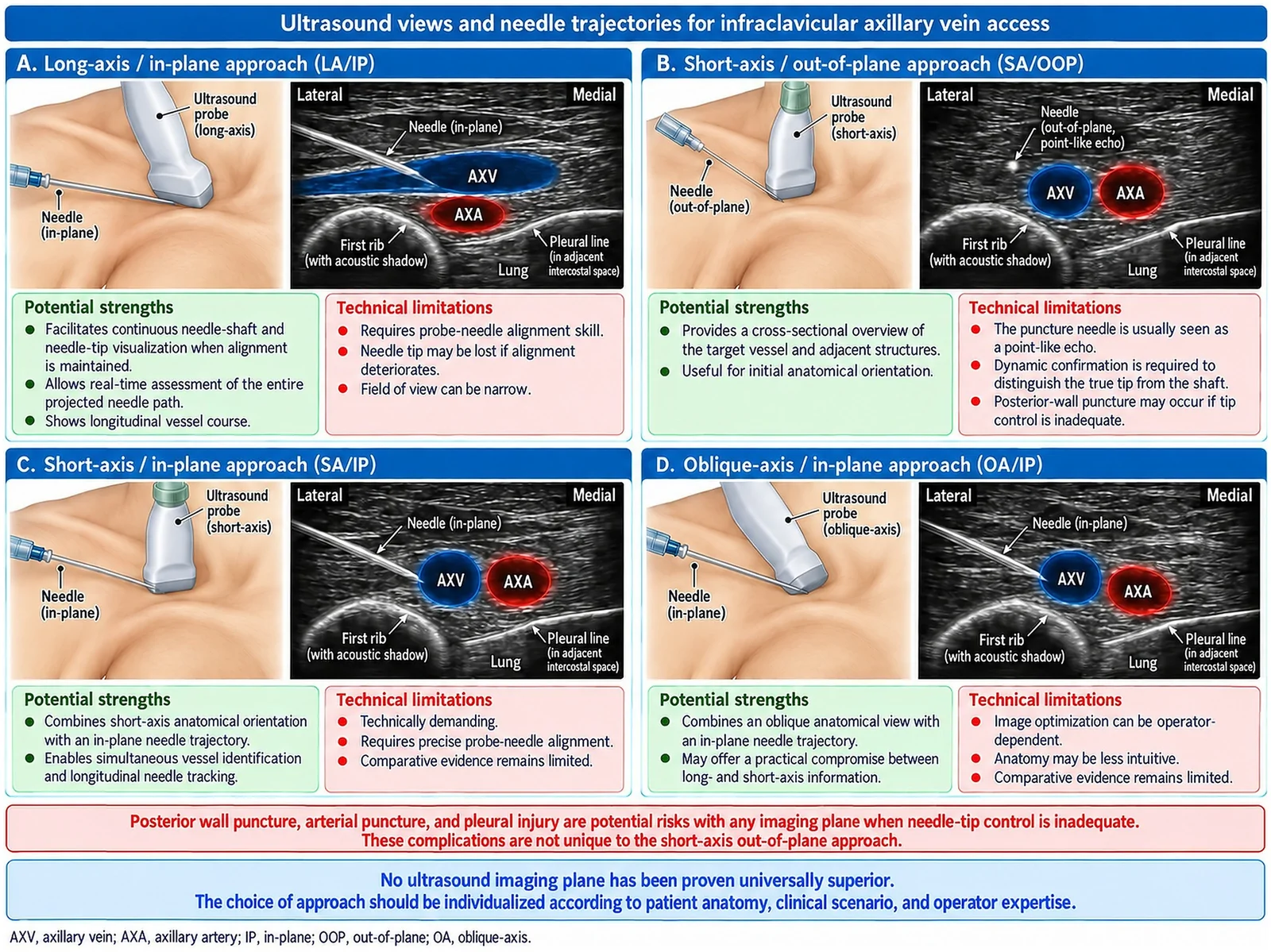
Ultrasound views and needle trajectories for infraclavicular axillary vein access. Panels **A–D** show **(A)** long-axis/in-plane (LA/IP), **(B)** short-axis/out-of-plane (SA/OOP), **(C)** short-axis/in-plane (SA/IP), and **(D)** oblique-axis/in-plane (OA/IP) approaches. Labels identify the ultrasound probe, needle and needle trajectory, lateral and medial orientation, axillary vein (AXV), axillary artery (AXA), first rib with acoustic shadow, pleural line in the adjacent intercostal space, and lung. The sonographic panels are schematic representations rather than patient-derived ultrasound images. LA/IP may facilitate continuous shaft and tip visualization when alignment is maintained; SA/OOP provides cross-sectional anatomical orientation but requires dynamic confirmation of the true needle tip. Comparative evidence for SA/IP and OA/IP remains limited. No ultrasound imaging plane has been proven universally superior. Posterior-wall puncture, arterial puncture, and pleural injury are potential risks with any imaging plane when needle-tip control is inadequate. AXA, axillary artery; AXV, axillary vein; IP, in-plane; LA, long-axis; OA, oblique-axis; OOP, out-of-plane; SA, short-axis.

## 6 Device selection and the complete insertion pathway

### 6.1 Device selection and device-specific pathways

‘AXV catheterization’ identifies a venous entry site, not a device category. Under the NAVIGATE nomenclature, a non-tunneled catheter inserted directly through the infraclavicular AXV into the central venous system is a centrally inserted central catheter (CICC). A catheter that is tunneled should be described as a tunneled CICC and further classified according to cuff status. A device connected to a subcutaneous chest reservoir is a chest TIVAD or chest port (34). A catheter is a PICC only when it enters through a peripheral upper-limb vein; tunneling a skin exit closer to the chest does not convert direct AXV access into a PICC.

Long-term device evidence comes mainly from oncology. Osawa and colleagues' historical comparison suggested that ultrasound-guided infraclavicular AXV access can avoid the classic clavicle-first-rib pinch-off pathway (35). Seo and colleagues reported 932 single-incision AXV TIVADs with 311,069 catheter-days of follow-up and provided long-term infection, thrombosis, migration, and device-dysfunction outcomes (11). OA/IP chest-port work and more recent single-incision or tunnelless chest-wall TIVAD studies further indicate that this access route can be integrated with patient comfort and cosmetic considerations (30, 36–38). Additional AXV chest-port studies support technical feasibility but are mainly single-center observational series or comparisons with mixed anatomical terminology, including studies by Lin, Seo, Hong, and Chen (39–42). These studies inform device feasibility and patient experience but do not establish long-term superiority of anatomically strict AXV access. The large O'Leary series included Broviac/Hickman catheters, ports, and dialysis catheters but did not provide causal comparative long-term infection or thrombosis data (43).

AXV access has also been used for temporary large-bore dialysis catheters. In a 29-patient pilot study of mechanically ventilated patients with acute kidney injury, Czarnik and colleagues reported 96.5% venipuncture success and 93.1% overall catheterization success (44). Large-bore dialysis catheters have different thrombosis and mechanical-risk profiles from standard CICCs, so this small study should not be interpreted as supporting routine replacement of IJV or femoral dialysis access.

### 6.2 Guidewire navigation and catheter-tip location

A key safety issue after AXV venipuncture is whether the guidewire and catheter follow the intended central venous pathway. In the 202-patient Czarnik series, there were six guidewire failures, one subcutaneous kink of a dialysis catheter, and 27 tip malpositions (27). In O'Leary's 2,586-attempt long-term-access series, 728 procedures (28.2%) required catheter manipulation under image intensification (43). In Shin and colleagues' 2024 randomized trial, the guidewire was intentionally not checked or corrected before catheter placement; in their author-defined infraclavicular SCV population, catheter malposition occurred in 10/224 (4.5%) left-sided versus 31/225 (13.8%) right-sided procedures (risk difference −9.3 percentage points, 95% CI −14.6 to −4.0; OR 0.29, 95% CI 0.14–0.61) (45). These findings inform guidewire and tip-management considerations but do not establish that left-sided AXV access is superior to right-sided AXV access (45). Collectively, these findings support treating venipuncture, guidewire navigation, catheter advancement, and final tip location as distinct but continuous safety endpoints.

Guidewire navigation can include ultrasound scanning of the ipsilateral IJV, the supraclavicular venous confluence, or other visible central venous segments. Nagalingam and colleagues routinely scanned the ipsilateral IJV and confluence after guidewire insertion and redirected wires that entered the IJV (23). The PANDA trial likewise confirmed wire position in the intended venous pathway before dilation (9). Such checks can identify an aberrant guidewire course before tissue dilation and catheter insertion, preventing blood return alone from being interpreted as completion of a safe access pathway. Accordingly, blood aspiration should be regarded only as supporting evidence of venous entry; real-time ultrasound confirmation of intraluminal needle-tip position and, where feasible, confirmation of the guidewire within the intended venous pathway should precede dilation (9, 23, 26).

Final catheter-tip location is a separate issue. For upper-body central venous access devices, contemporary guidance recognizes the lower third of the superior vena cava, the cavoatrial junction, or the high right atrium as appropriate tip regions, with the final target and confirmation method depending on device type and clinical context (26). Chest radiography, fluoroscopy, intracavitary electrocardiography, transthoracic ultrasound saline/bubble testing, and rapid infusion ultrasonography all have potential roles (26, 46–50). In 70 patients undergoing successful author-defined right axillary venous catheterization, Miao and colleagues reported rapid infusion ultrasonography sensitivity of 100% and specificity of 77.8%, with shorter assessment time than chest radiography, but the study did not strictly define the first-rib segment and used chest radiography as the reference standard (50). This is insufficient to establish universal replacement of other tip-location methods.

The SIC-Port protocol integrates RaCeVA, ultrasound-guided puncture, pleural ultrasound, tip navigation, intracavitary ECG and/or transthoracic echocardiographic bubble testing into a chest-port insertion bundle (48). It is a valuable example of pathway standardization, but the bundle itself is not a randomized-trial-validated guideline and is therefore treated here as an evidence-informed implementation example rather than a proven superior package.

## 7 Clinical and comparative outcomes

### 7.1 AXV versus IJV

In the 2023 two-center randomized trial by Czarnik and colleagues, 614 mechanically ventilated critically ill patients were randomized and 610 completed the study. AXV versus IJV venipuncture success was 99.7% versus 100%, first-pass success 74.1% versus 79.9%, catheterization success 96.7% versus 98.7%, catheter-tip malposition 10.1% versus 9.9%, and early mechanical complications 2.6% versus 3.0%, with no statistically significant differences (13). Shinde and colleagues' randomized cardiac-surgery study likewise suggested similar short-term success between the two ultrasound-guided access sites (51). Fournil and colleagues' open-label pilot explicitly used a descriptive design without formal between-group inference; procedural overall success was 96/100 (96.0%) for distal (low) IJV versus 90/101 (89.1%) for AXV, and first-pass success was 90.0% versus 80.2% (52). Late colonization/infection analyses had 24 and 28 missing observations, and the published overall-complication row was internally inconsistent; only clearly defined component outcomes were retained.

The 2026 systematic review by Królicki and colleagues included three randomized AXV-versus-IJV trials (*n* = 908) in its primary meta-analysis. Overall catheterization success had RR 0.98 (99% CI 0.95–1.02), first-pass success RR 0.93 (99% CI 0.88–1.00), and cumulative mechanical complications RR 1.05 (95% CI 0.69–1.61) (12). For first-pass success, the point estimate favored IJV and the 99% CI reached the null (upper bound 1.00); in the absence of an independently interpretable prespecified non-inferiority or equivalence margin, this result should not be interpreted as evidence of equivalence. Trial sequential analysis reached the authors' optimal information size, but the article did not provide a numerical equivalence margin that could be independently transcribed. A subsequent methodological correspondence further cautioned that the three underlying trials differed in ultrasound needle-visualization technique, cannulation laterality, and patient positioning, introducing clinically relevant methodological heterogeneity that may not be captured by statistical heterogeneity testing alone (53). We therefore describe currently studied short-term overall success and early mechanical outcomes as broadly similar rather than independently declaring equivalence. This inference cannot be extended to long-term infection, thrombosis, all device types, or all operator environments.

### 7.2 AXV strategy versus landmark-guided SCV strategy

Zhou and colleagues' 2022 meta-analysis included five randomized trials (*n* = 1,852). Compared with landmark-guided SCV access, an ultrasound-guided AXV strategy had higher first-pass puncture success (four trials, *n* = 1,792; RR 1.17, 95% CI 1.13–1.22). Overall success in the initial four-study analysis was heterogeneous (*n* = 1,720; RR 1.07, 95% CI 0.98–1.17; I^2^ = 85%); after exclusion of the Xu study, sensitivity analysis yielded RR 1.09 (three trials, *n* = 351; 95% CI 1.04–1.15; I^2^ = 0%). Arterial puncture (four trials, *n* = 1,752; RR 0.18, 95% CI 0.06–0.55) and pneumothorax/hemothorax (five trials, *n* = 1,852; RR 0.12, 95% CI 0.02–0.64) were less frequent (54). However, this comparison simultaneously changes both anatomical target and imaging guidance. The appropriate inference is that the ultrasound-guided AXV strategy outperformed the landmark-guided SCV strategy, not that AXV anatomy itself is intrinsically superior to a true SCV target.

Other systematic reviews and randomized studies comparing supraclavicular with infraclavicular/subclavian/axillary ultrasound strategies likewise show that first-pass success, procedure time, and mechanical outcomes vary with access definition, imaging strategy, and operator experience (55–58). Because anatomical localization was not consistently defined relative to the first rib, these studies are best interpreted as contextual comparative evidence rather than direct estimates of an AXV-specific site effect (55–58).

### 7.3 Real-world feasibility

Real-world series support the feasibility of AXV catheterization but are not suitable for causal site comparisons. O’Leary and colleagues reported 2,572 successes among 2,586 ultrasound-guided long-term-access attempts (99.5%); because conversions included AXV-to-IJV, landmark SCV, external jugular, and contralateral AXV access, the 1,923 final AXV placements are not a valid denominator for the initial AXV strategy (43). In 202 mechanically ventilated critically ill patients, Czarnik and colleagues reported 95.1% complete catheterization success (95% CI 91.1–97.6) (27). Kosiński and colleagues reported 96.7% success across 734 LA/IP attempts (59), and Glen and colleagues reported 94% success in a mechanically ventilated ICU cohort (28). Center experience, patient selection, and outcome definitions continue to limit external validity.

Key comparative studies and adjacent technical evidence are summarized in Supplementary Table S4; the main text emphasizes findings that materially affect clinical interpretation.

## 8 Complications and risk mitigation

### 8.1 Arterial puncture, pneumothorax, and neural injury

Direct AXV series show that arterial puncture and pneumothorax are uncommon but not absent. Czarnik's prospective ICU series and the 6-year Kosiński experience reported arterial puncture rates in the low single-digit range, and Kosiński reported pneumothorax in 0.14% of procedures (27, 59). Adjacent neural structures may also be contacted during needle advancement, and rare brachial-plexus events have been reported (59). Risk mitigation should therefore emphasize identification of the pleura and adjacent structures, continuous needle-tip confirmation, minimization of ineffective puncture attempts, and early abandonment or change of target when visualization remains inadequate. Contemporary multicenter data from ultrasound-guided central venous catheterization also associate multiple punctures and operator experience with major mechanical complications (60).

### 8.2 Guidewire and catheter malposition

Malposition is reported more consistently than pneumothorax or arterial puncture across multiple AXV studies (13, 23, 27, 59, 61, 62). In the Kosiński series, one of the most common early problems was catheter passage into the ipsilateral IJV or another non-ideal vein (59), and the severe-trauma cohort reported by Gu and colleagues likewise highlighted catheter malposition and suboptimal tip position (62). A safety bundle for AXV access therefore needs to include guidewire pathway assessment and final tip-location confirmation, not ultrasound only during venipuncture.

### 8.3 Pinch-off syndrome and mechanical pathway

Classic landmark-guided subclavian access may place a catheter within the costoclavicular compression zone. Osawa and colleagues compared 100 landmark-guided procedures with 107 ultrasound-guided infraclavicular AXV procedures; all ultrasound-guided placements were pinch-off grade 0 at insertion and showed no progression during follow-up, whereas the landmark group included clinically relevant pinch-off grades and progression (35). This supports the mechanical rationale that a more lateral, intravascular extrathoracic entry pathway can avoid costoclavicular compression. Because the study was a historical comparison and changed both access route and ultrasound use, the full effect cannot be attributed to the AXV site itself.

### 8.4 Infection: event rates exist, but a causal advantage is unproven

AXV-associated infectious outcomes have been reported, but the reported endpoints are not interchangeable. Kosiński and colleagues reported CRBSI in 2.8% of successful catheterizations, corresponding to 0.23 per 1,000 line-days, in their 6-year retrospective series (59). Seo and colleagues reported 23 suspected catheter-related bloodstream infections and 4 pocket infections among 932 TIVADs during 311,069 device-days of follow-up, with a median indwelling time of 467 days (11). In the severe-trauma cohort, Gu and colleagues reported CRBSI at 3.38 per 1,000 catheter-days during the trauma ICU stay (62). Fournil and colleagues reported no catheter-related infection in the AXV group, although late infection assessment was affected by missing follow-up data (52). Suspected CRBSI, pocket infection, and CRBSI incidence rates were therefore retained as distinct outcomes rather than combined into a common infection-rate range. Differences in definitions, denominators, device types, observation periods, and ascertainment also preclude any conclusion that AXV access independently reduces infection compared with IJV or other central venous routes.

The traditional lower-infection signal associated with 'subclavian' access largely derives from landmark-era and site-labeled studies (1, 2). Exit-site location, device type, disease severity, maximal sterile barriers, dressing practices, and maintenance systems all influence CRBSI (63). The 2026 meta-analysis explicitly noted that whether an SCV infection advantage transfers to AXV access has not been directly examined (12). We therefore do not make a causal claim that a chest-wall exit site or AXV entry independently lowers infection risk.

### 8.5 Thrombosis: the net effect remains unresolved

Catheter-related thrombosis and overall venous thromboembolism should be considered separately because they represent different outcome constructs. In the 6-year retrospective Kosiński series, catheterized-vein thrombosis occurred in fewer than 1% of 710 successful catheterizations; all reported events were left-sided and associated with non-ideal brachiocephalic tip location (59). Seo's long-term TIVAD cohort reported a small number of symptomatic central venous stenosis/thrombosis events during 311,069 device-days of follow-up (11). In a 2025 non-randomized breast-cancer TIVAD comparison, six-month thrombosis outcomes were broadly similar between AXV and IJV access (38). These studies used different device populations, follow-up periods, and ascertainment approaches and do not establish either a protective or harmful independent effect of AXV access on CRT.

CRT is multifactorial and can be understood as the interaction of catheter-related endothelial injury, local flow disturbance or stasis, and patient-level hypercoagulability. Catheter caliber and vessel occupancy, catheter length and insertion side, repeated vascular trauma, and non-ideal tip position may modify local thrombotic conditions, whereas malignancy, major trauma, systemic inflammation, immobility, critical illness, and mechanical ventilation may increase baseline thrombotic susceptibility (11, 38, 59, 62). More broadly, statins have been reported to modulate several thrombotic pathways, including tissue-factor expression, endothelial thrombomodulin, thrombin generation, and platelet activation; however, this mechanistic evidence is not specific to catheter-related thrombosis or AXV access and should not be interpreted as evidence that statin therapy modifies AXV-associated CRT risk (64).

Overall VTE represents a separate patient-level outcome. Gu and colleagues reported VTE in 27.3% of 132 severely injured trauma patients during the trauma ICU stay, including deep-vein thrombosis in 25.8% and pulmonary embolism in 6.8%; VTE was identified using lower-limb venous ultrasound or CT pulmonary angiography and was not restricted to catheter-related thrombosis (62). The study further identified older age, fever, and longer mechanical-ventilation duration as factors associated with VTE, underscoring the importance of baseline patient risk in this population. The 27.3% estimate should therefore not be interpreted as an AXV-specific CRT rate. Future AXV studies should report CRT separately from overall VTE and prespecify catheter characteristics, insertion side, tip location, baseline VTE risk, observation period, and whether thrombosis ascertainment is symptom-triggered or based on protocolized ultrasound surveillance. The principal complication domains, direct evidence, risk-control strategies, and residual uncertainties are summarized in Table 3.

**Table 3:** Major complications, direct AXV evidence, risk control, and residual uncertainty.

Complication	Direct AXV evidence	Major risk factors/confounding	Risk-control approach	Residual uncertainty
Arterial puncture	Approximately 0.7%–2.5% in large/prospective series (27, 59)	Loss of needle tip, deep target, artery-vein overlap	Continuous needle-tip confirmation; change target/plane when required	True differences among techniques remain uncertain
Pneumothorax/hemothorax	Low but non-zero; pneumothorax 0.14% in Kosiński series (59)	Pleura close to target, steep needle path, poor image quality	Pre/intraprocedural pleural identification; limit ineffective attempts	Very rare events require large comparative datasets
Brachial plexus/neural injury	Rare; reported in real-world series (59)	Lateral target, adjacent neural structures, blind deep advancement	Identify adjacent structures; stop with unexpected radiating pain	Neurological outcomes are underreported
Guidewire/tip malposition	Repeatedly reported across studies (13, 23, 27, 59, 61, 62)	Side, position, J-tip orientation, catheter length	Guidewire navigation plus final tip-location confirmation	Best combination of navigation methods not established
CRBSI	Kosiński: CRBSI 2.8% (0.23/1,000 line-days) (59); Seo: suspected CRBSI and pocket infection during long-term TIVAD follow-up (11); Gu: CRBSI 3.38/1,000 catheter-days during trauma ICU stay (62); Fournil: no catheter-related infection in the AXV arm, with incomplete late follow-up (52).	Strong confounding by patient, device, exit site, and maintenance	Maximal sterile barrier, maintenance bundle, remove unnecessary catheters	Definitions, denominators, follow-up, and ascertainment differ across studies; whether AXV is superior to IJV remains unknown.
Catheter-related thrombosis (CRT)	Kosiński: catheterized-vein thrombosis <1% among 710 successful cannulations in a 6-year retrospective series; reported events were left-sided and associated with non-ideal brachiocephalic tip location (59). Seo: one early AXV thrombosis and four late symptomatic central-vein stenosis/thrombosis events during 311,069 device-days (11).	Catheter caliber and vessel occupancy, endothelial injury, insertion side and catheter length, non-ideal tip position, malignancy and baseline VTE risk	Optimize catheter size and tip position; individualize device and side selection; use ultrasound evaluation when clinically indicated	Observation periods and ascertainment methods differed, and protocolized thrombosis surveillance was not consistent across studies; the independent effect of AXV access on CRT remains unresolved.
Overall venous thromboembolism (VTE)	VTE 27.3% in 132 severe-trauma patients during trauma ICU stay; this was an overall VTE outcome, not a catheter-specific CRT rate (62). VTE was identified using lower-limb venous ultrasound or CT pulmonary angiography.	Severe trauma, immobility, inflammation, mechanical ventilation, baseline VTE risk	Standard VTE assessment and prevention according to clinical context	Should not be interpreted as an AXV-specific CRT rate.
Pinch-off	Lateral AXV entry can avoid a compressive costoclavicular course (35)	Costoclavicular passage	Avoid classic compression pathway	Long-term comparisons across port/catheter materials are limited

## 9 Training and clinical implementation

### 9.1 Training, competency, and quality indicators

AXV access requires familiarity with three-dimensional infraclavicular anatomy and reliable needle-tip control. In a single-operator LC-CUSUM study, Zhu and colleagues analyzed 65 patients and observed a marked improvement in procedure time and first-pass success after approximately the 29th case; first-pass success was 27.6% in the early phase and 96.8% in the later phase (14). The study demonstrates an important learning effect but does not establish ‘29 cases’ as a universal minimum. Prior ultrasound skill, mentoring, patient complexity, technique, and the definition of success all influence the learning curve.

International vascular-access training consensus favors a sequence of theory, simulation, supervised clinical practice, and performance-based authorization rather than a simple procedure count (65). Simulation, case-based learning, and self-directed learning-curve research further show that training design and assessment metrics affect skill acquisition, although the populations and settings are not identical to routine critical care (66–68). PERSEUS, the 2025 American Society of Echocardiography recommendations, the 2025 Association of Anaesthetists guideline, the Society of Hospital Medicine position statement, and the American Society of Anesthesiologists guideline emphasize competence in ultrasound anatomy, needle-tip tracking, guidewire confirmation, aseptic technique, and complication recognition before independent practice (26, 46, 69–71). AXV-specific training should additionally include first-rib anatomy, dynamic target selection, alternative imaging planes, guidewire and tip management, pleural assessment, and rescue strategies after failure.

Team-level quality measurement should extend beyond overall success. Useful indicators include first-pass success, number of punctures, conversion to another site, guidewire/catheter malposition, arterial puncture, pneumothorax, non-ideal tip location, CRBSI per 1,000 catheter-days, symptomatic and surveillance-detected CRT, and patient experience. Such measures distinguish a technique that can be completed from a pathway that is reliably safe.

### 9.2 Evidence-informed clinical implementation framework

Based on the available evidence, we propose an externally unvalidated evidence-informed clinical implementation framework. Clinical indication and device choice are informed mainly by device terminology and vascular-access consensus (34, 47). Systematic ultrasound assessment and identification of a target lateral to the first rib are supported by anatomy, RaCeVA, and direct AXV studies (3–9, 16–18, 20, 34, 72). Positioning and needle-tip strategies are informed by randomized and observational technical studies (10, 21–24, 29–33, 73–75). Guidewire navigation, tip location, and immediate complication exclusion draw on AXV studies, SIC-Port, and vascular-ultrasound guidance (13, 25–27, 45, 46, 48–50, 59, 62, 76), while securement, dressing, and maintenance derive largely from general CVC guidance and consensus (26, 47, 63, 71). Because the directness and study design vary across nodes, the framework must not be presented as a validated clinical guideline or standard of care.

The framework is conceptually consistent with RaCeVA, SIC-Port, and contemporary critical-care and safe-vascular-access consensus (20, 26, 47, 48). It links patient/device goals, ultrasound anatomy, target selection and position, puncture strategy, venous-entry confirmation, guidewire navigation, catheter advancement and tip location, immediate complication exclusion, and securement/maintenance, with exit or escalation options when anatomy, needle visualization, or guidewire behavior is unfavorable.

Figure 4 presents the proposed evidence-informed clinical implementation framework and explicitly distinguishes evidence directness from evidence quality.

**Figure 4:**
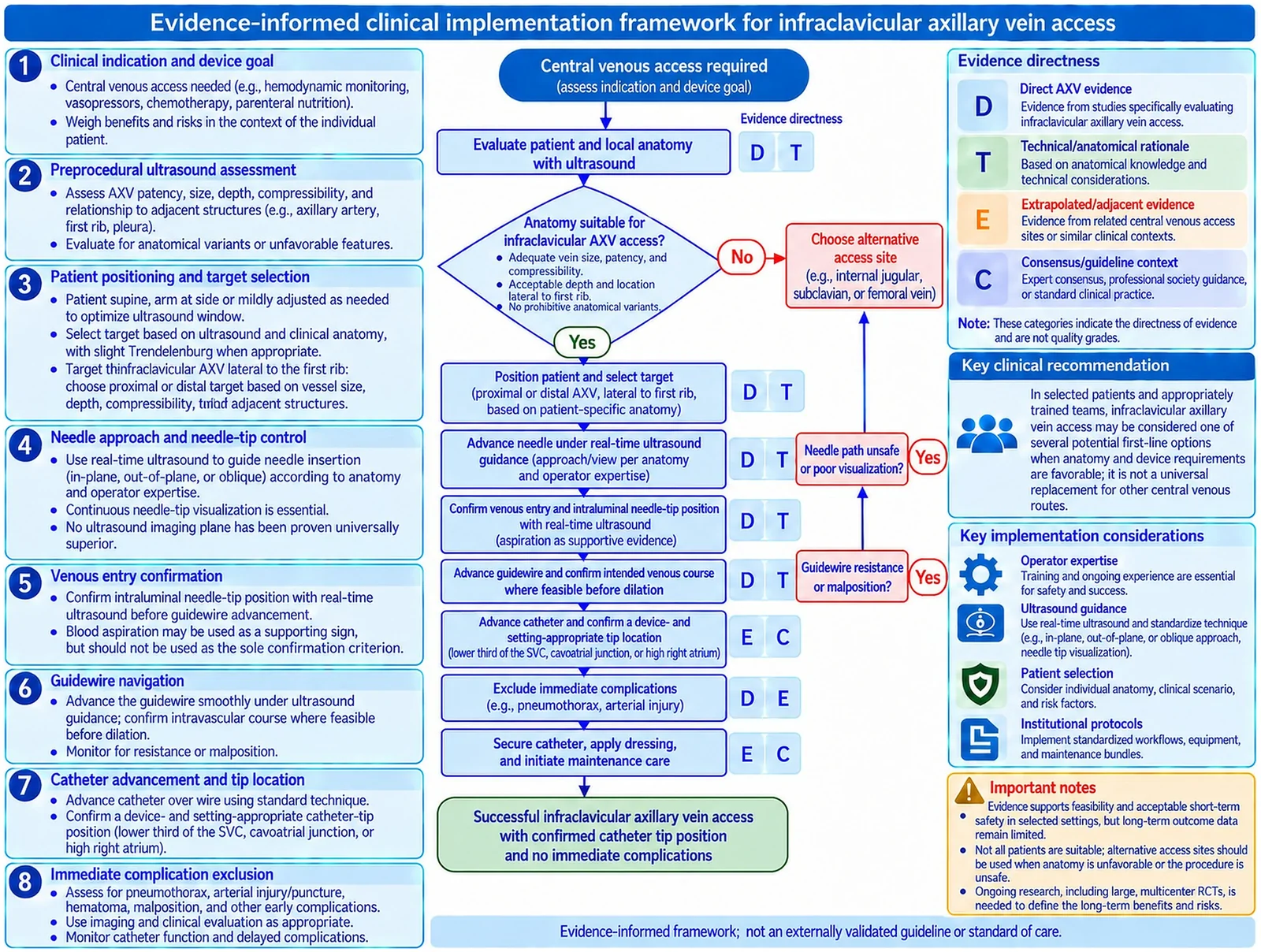
Evidence-informed clinical implementation framework for infraclavicular axillary vein access. The text-based framework integrates clinical indication and device goals, preprocedural ultrasound, patient positioning and target selection, needle approach and needle-tip control, venous-entry confirmation, guidewire navigation, catheter advancement and tip location, immediate complication exclusion, and securement/maintenance. D, T, E, and C indicate evidence directness rather than evidence quality: D, direct AXV clinical evidence; T, technical/anatomical rationale; E, extrapolated/adjacent evidence; C, consensus/guideline context. The framework includes exit or escalation pathways for unfavorable anatomy, unsafe needle visualization, or guidewire resistance/malposition and has not undergone external validation; it is not a clinical guideline or standard of care.

## 10 Discussion

### 10.1 Clinical positioning and net-benefit interpretation

Taken together, the evidence supports a conditional role for infraclavicular AXV access as one of several potential first-line options in selected patients and trained teams, rather than as a universal replacement for other central venous routes. Randomized trials and the recent meta-analysis suggest broadly similar short-term overall procedural success and early mechanical outcomes versus IJV access, without independently establishing equivalence (12, 13, 51). Between-trial differences in needle-visualization technique, cannulation laterality, and patient positioning further limit route-level generalization (53). Potential advantages related to extrathoracic entry and chest-wall device pathways should therefore be interpreted within the broader context of device type, operator expertise, and downstream catheter management (11, 35, 38).

Access-site selection should therefore be individualized according to patient anatomy, device requirements, operator expertise, and maintenance capability. AXV access is reasonable when the infraclavicular window is favorable and the team can maintain needle-tip control and manage guidewire and tip position; otherwise, an alternative route should be selected.

A central contribution of this review is to treat successful venipuncture as an intermediate event rather than the endpoint of AXV access. In several studies, clinically relevant problems occurred after the needle entered the vein, when the guidewire or catheter deviated from the intended central route (13, 23, 27, 59, 61, 62). Evaluation and training should therefore move from a needle-to-vein success model to a complete-pathway safety model in which anatomical definition, dynamic ultrasound, puncture, guidewire navigation, tip location, immediate complication assessment, securement, and long-term maintenance form a continuous safety chain.

### 10.2 Unresolved controversies

#### 10.2.1 Infection

Infection risk should not be used as a stand-alone reason to prefer AXV access; device type, dwell time, patient characteristics, and maintenance practices remain important determinants (11, 52, 59, 62, 63).

#### 10.2.2 Thrombosis

Access-site choice should likewise not be based on an assumed CRT advantage; catheter characteristics, endothelial injury, tip position, and baseline thrombotic risk should be integrated into device and site selection (11, 38, 59, 62, 64).

#### 10.2.3 Catheter-to-vein ratio

No evidence supports direct transfer of a fixed PICC-derived catheter-to-vein ratio threshold to infraclavicular AXV CICC placement. Such ratios may remain physiologically plausible research variables; future studies should model catheter outer diameter relative to AXV cross-sectional area as a continuous exposure and relate it to prospectively screened CRT.

#### 10.2.4 Patient selection

AXV access may be particularly useful when neck access is undesirable, such as in patients with tracheostomy, neck surgery or injury, selected mechanically ventilated patients, and those requiring long-term chest-wall devices (27, 28, 38, 44, 59, 61, 62). Bleeding risk requires a more nuanced assessment rather than being treated as an isolated indication for AXV access. Contemporary vascular-access guidance recommends that patients with coagulopathy or receiving anticoagulant therapy be assessed according to the type and size of the device, procedural complexity, vascular anatomy, operator experience, and the feasibility of ultrasound-guided insertion and local compression; the preferred access strategy should therefore be individualized rather than determined by bleeding risk alone (26). The PANDA trial demonstrated the feasibility of ultrasound-guided AXV catheterization in cardiac-surgical patients considered susceptible to bleeding, but it compared proximal and distal AXV approaches and did not establish that AXV access itself reduces bleeding relative to IJV or other central venous routes (9). Bleeding risk may also become relevant after catheter placement because symptomatic CRT or other VTE may require therapeutic anticoagulation. Recent RIETE registry data identified patient-level associations with bleeding during anticoagulant treatment for acute VTE, iincluding an association between lower low-density lipoprotein cholesterol levels and bleeding risk; however, these findings were not specific to catheter-related thrombosis, vascular-access procedures, or AXV cannulation (77). Accordingly, thrombotic and bleeding risks should be considered together with anatomy, device requirements, and operator expertise when selecting the vascular-access pathway, without assuming a bleeding-risk advantage for AXV access.

#### 10.2.5 Operator effect

The Zhu learning curve, Maddali imaging-plane comparison, and Kåsine needle trial suggest that operator-technique interaction can materially affect outcomes (10, 14, 32). In the ultrasound era, differences attributed to access site may be partly modified by needle-tip control, guidewire management, and the ability to convert safely after failure. Future trials should prespecify operator experience as a stratification or effect-modification variable.

#### 10.2.6 Imaging-plane selection

Technique should be individualized to patient anatomy and the operator's ability to maintain reliable needle-tip control rather than protocolized around a single preferred plane (10, 29–31, 73).

### 10.3 Research priorities

Priority research areas are: (1) standardized AXV/SCV nomenclature based on the lateral border of the first rib and reproducible sonographic landmarks, including inter-operator reliability; (2) a core outcome set that standardizes first-pass success, site success, overall success, puncture number, access time, cannulation time, guidewire malposition, tip malposition, and mechanical complications; (3) pragmatic multicenter comparisons of proximal versus distal targets and LA/IP, SA/OOP, SA/IP, and OA/IP after standardized anatomical definitions, ideally with blinded video assessment of needle-tip quality; (4) prospective long-term cohorts or randomized studies reporting CRBSI by catheter-days and using prespecified ultrasound surveillance for symptomatic and asymptomatic CRT, catheter function, and device survival; (5) patient-centered outcomes including pain, neck/arm mobility, sleep, daily activity, cosmetic satisfaction, and quality of life, especially for TIVADs; (6) health-economic analyses incorporating disposables, ultrasound resources, procedure time, radiography/fluoroscopy, conversion after failure, and avoided complications; (7) validated learning-curve and performance-based competency tools rather than a single fixed case count; and (8) multicenter registries using standardized terminology and outcomes to detect rare mechanical events and define long-term net benefit across patient subgroups.

### 10.4 Limitations

This review used a targeted evidence-informed narrative approach. Two reviewers independently screened titles/abstracts and independently performed the secondary relevance and evidence-role assessment of retained records. Nevertheless, no formal risk-of-bias instrument, GRADE framework, or preregistered protocol was used. Because the evidence base spans heterogeneous anatomical, technical, device, comparative, and contextual sources, evidence-role and directness judgments remain partly interpretive even with dual independent screening. The 24 supplementary/contextual evidence documents comprised 21 identified through guideline searches, citation tracking, or broader central-venous-access context and three added during revision. These documents are therefore reported separately from the 52 documents linked to the core three-database search.

Historical inconsistency in AXV/SCV terminology sometimes prevented confident localization relative to the first rib; such sources were conservatively treated as indirect evidence, which may have underestimated the amount of truly AXV-specific evidence.

Full articles were not available for every historical source or conference record. Verification level was documented source by source, and only records for which the full article was actually obtained were described as full-text audited; formal abstracts or metadata support only what they explicitly report. Some technical recommendations are necessarily derived from general CVC guidelines, device consensus, or indirect evidence and should not be regarded as AXV-specific findings. Finally, many AXV studies arise from experienced single centers or selected populations, and some reports may represent expanded or overlapping cohorts. Explicitly overlapping reports were grouped under a shared study family, but undisclosed sample overlap and operator effects cannot be completely excluded.

## 11 Conclusion

The infraclavicular AXV, defined by the lateral border of the first rib and assessed dynamically with ultrasound, represents a feasible central venous access route in selected patients and experienced teams. Current evidence supports considering AXV access as one potential first-line option when anatomy, device requirements, operator expertise, and downstream catheter management are favorable, but not as a universal replacement for IJV or SCV access. Long-term infection and thrombosis outcomes, the optimal imaging strategy, AXV-specific catheter-to-vein thresholds, and the patient groups with greatest net benefit remain uncertain and require further evaluation using standardized anatomical definitions and complete-pathway safety outcomes.
