# Supplementary material for: Ultrasound-guided infraclavicular axillary vein access for central venous catheterization: an evidence-informed narrative review
Source: Front Med (Lausanne). 2026 Oct 28;13:1999229. doi: 10.3389/fmed.2026.1999229 (PMC13753970; doi:10.3389/fmed.2026.1999229)
Supplement: Supplementary file 1 [file Table1.docx]

**Supplementary Table S1. Formal database search strategies**

The strings below reproduce the searches as executed. No language restriction was applied. The three database searches were completed on 6 August 2026.

| **Database** | **Coverage/search date and limits** | **Exact search strategy as executed** | **Records identified (n)** |
| --- | --- | --- | --- |
| PubMed/MEDLINE | Inception to 2026-08-06; no language limit | (  "Axillary Vein"[Mesh]  OR "axillary vein"[tiab]  OR "axillary venous"[tiab]  OR "infraclavicular vein"[tiab]  OR "infraclavicular venous"[tiab]  OR "infraclavicular subclavian"[tiab]  OR "proximal axillary"[tiab]  OR "distal axillary"[tiab]  OR transpectoral[tiab] ) AND (  "Catheterization, Central Venous"[Mesh]  OR "Vascular Access Devices"[Mesh]  OR "central venous catheter"[tiab]  OR "central venous catheters"[tiab]  OR "central vein catheter"[tiab]  OR "central vein catheters"[tiab]  OR "central venous access"[tiab]  OR "central vein cannulation"[tiab]  OR "central venous cannulation"[tiab]  OR "venous access port"[tiab]  OR "venous access ports"[tiab]  OR "implantable port"[tiab]  OR "implantable ports"[tiab]  OR TIVAD[tiab]  OR TIVADs[tiab]  OR CICC[tiab]  OR "dialysis catheter"[tiab]  OR "dialysis catheters"[tiab]  OR "cardiac implantable electronic device"[tiab]  OR "cardiac implantable electronic devices"[tiab] ) AND (  "Ultrasonography, Interventional"[Mesh]  OR ultrasonograph*[tiab]  OR ultrasound[tiab]  OR sonograph*[tiab]  OR "real time"[tiab]  OR "real-time"[tiab] ) | 162 |
| Embase | Inception to 2026-08-06; no language limit | ('axillary vein'/exp OR 'axillary vein':ti,ab,kw OR 'axillary venous':ti,ab,kw OR 'infraclavicular vein':ti,ab,kw OR 'infraclavicular venous':ti,ab,kw OR 'infraclavicular subclavian':ti,ab,kw OR 'proximal axillary':ti,ab,kw OR 'distal axillary':ti,ab,kw OR transpectoral:ti,ab,kw) AND ('central venous catheterization'/exp OR 'vascular access device'/exp OR 'central venous catheter':ti,ab,kw OR 'central venous catheters':ti,ab,kw OR 'central vein catheter':ti,ab,kw OR 'central vein catheters':ti,ab,kw OR 'central venous access':ti,ab,kw OR 'central vein cannulation':ti,ab,kw OR 'central venous cannulation':ti,ab,kw OR 'venous access port':ti,ab,kw OR 'venous access ports':ti,ab,kw OR 'implantable port':ti,ab,kw OR 'implantable ports':ti,ab,kw OR tivad:ti,ab,kw OR tivads:ti,ab,kw OR cicc:ti,ab,kw OR 'dialysis catheter':ti,ab,kw OR 'dialysis catheters':ti,ab,kw OR 'cardiac implantable electronic device':ti,ab,kw OR 'cardiac implantable electronic devices':ti,ab,kw) AND ('ultrasound guided procedure'/exp OR ultrasonograph*:ti,ab,kw OR ultrasound:ti,ab,kw OR sonograph*:ti,ab,kw OR 'real time':ti,ab,kw) | 302 |
| Web of Science Core Collection | All editions; through 2026-08-06; all languages | TS=( ( "axillary vein" OR "axillary venous" OR "infraclavicular vein" OR "infraclavicular venous" OR "infraclavicular subclavian" OR "proximal axillary" OR "distal axillary" OR transpectoral ) AND ( "central venous catheter*" OR "central vein catheter*" OR "central venous access" OR "central vein cannulat*" OR "central venous cannulat*" OR "venous access port*" OR "implantable port*" OR TIVAD* OR CICC OR "dialysis catheter*" OR "cardiac implantable electronic device*" ) AND ( ultrasonograph* OR ultrasound OR sonograph* OR "real time" OR "real-time" ) ) Indexes: Web of Science Core Collection (SCI-EXPANDED and ESCI) Timespan: All years Language: All languages Document types: All (screened during selection) | 163 |

**Note.** Embase returned 302 platform records, comprising 273 bibliographic article records and 29 ClinicalTrials.gov registry records. The 29 registry records were separated before bibliographic deduplication; therefore, 273 Embase bibliographic records entered deduplication. Across the three databases, 598 bibliographic records entered deduplication (162 PubMed/MEDLINE + 273 Embase + 163 Web of Science Core Collection).
