# Supplementary material for: Ultrasound-guided infraclavicular axillary vein access for central venous catheterization: an evidence-informed narrative review
Source: Front Med (Lausanne). 2026 Oct 28;13:1999229. doi: 10.3389/fmed.2026.1999229 (PMC13753970; doi:10.3389/fmed.2026.1999229)
Supplement: Supplementary file 2 [file Table3.docx]

**Eligibility and evidence-selection boundaries**

| **Eligible / potentially retainable evidence** | **Not selected or excluded from the core AXV evidence set** |
| --- | --- |
| • Human clinical studies; healthy-volunteer or anatomical imaging studies; AXV-specific simulation/training studies; systematic reviews; guidelines; and consensus documents.  • Ultrasound-guided infraclavicular AXV, proximal/distal AXV, or an “infraclavicular SCV” that could be anatomically reinterpreted from reproducible landmarks.  • Relevant device pathways included CICCs, tunneled catheters, TIVADs, dialysis catheters, and selected technically transferable cardiac-device access studies.  • No date or language restriction was applied. | • Axillary-artery studies; thrombosis-diagnosis or arteriovenous-fistula surveillance studies without CVC puncture relevance.  • Peripheral/midline/PICC studies without direct AXV relevance and non-target device/access pathways.  • Landmark- or venography-only studies without a relevant ultrasound comparator, and reports without extractable evidence.  • Studies labeled only “subclavian,” “AxV/SCV,” or “axillary” without the first rib, infraclavicular location, or another reproducible landmark were not automatically classified as direct AXV evidence. |

**Supplementary Table S3. Secondary relevance and core-evidence selection accounting**

The 183 records retained after title/abstract screening underwent an independent secondary relevance and evidence-role assessment by the same two reviewers. The assessment considered clinical relevance, anatomical directness, technical transferability, and redundancy; disagreements between the two reviewers were adjudicated by a third reviewer. These categories describe narrative-review evidence selection and redundancy management; they are not PRISMA-style full-text exclusion reasons and do not imply that all 131 reports were retrieved and assessed in full.

| **Stage / primary disposition** | **n** | **Interpretation** |
| --- | --- | --- |
| Records entering secondary relevance/evidence-role assessment | 183 | Records retained after title/abstract screening. |
| Core database-derived evidence documents retained | 52 | Retained as the core database-derived evidence set; source-level Screen_ID cross-mapping, where available, is reported in Supplementary Table S2. |
| Records not selected for the core evidence matrix | 131 | Assigned one primary disposition category below. |
| NS1: Overlapping or earlier report | 5 | A more complete or representative report was used. |
| NS2: Direct/highly relevant AXV evidence with limited incremental contribution | 21 | Relevant evidence was not retained in the core matrix when the same point was represented by stronger or more complete sources. |
| NS3: CIED/electrophysiology-adjacent evidence | 38 | Technically adjacent evidence outside the core central-venous-catheter pathway; only selected highly transferable sources were retained. |
| NS4: Insufficient AXV/SCV anatomical localization | 24 | First-rib boundary or another reproducible anatomical landmark was insufficient to treat the record as direct AXV core evidence. |
| NS5: Non-target access route or device | 5 | For example, PICC, ECC, or other non-target peripheral/upper-limb access pathways. |
| NS6: Secondary, guideline, or general-CVC source with limited additional AXV-specific information | 17 | Could inform context but did not add sufficient AXV-specific information for the core database-derived matrix. |
| NS7: Auxiliary or downstream question | 12 | Primarily addressed tip confirmation, complication detection, coatings, preprocedural venography, or other downstream/adjunct questions. |
| NS8: Insufficient source level for the core matrix | 9 | Protocol, letter, case report, conference abstract, or metadata-only source; not treated as a formal full-text exclusion. |
| Supplementary/contextual evidence documents included | 24 | 21 identified through citation tracking, guideline searches, or broader central-venous-access context in the original review, plus 3 contextual/methodological sources added during revision in response to reviewer comments. |
| Final evidence documents | 76 | 52 core-search + 24 supplementary/contextual documents. |
